# An explorative analysis of the differences in levels of happiness between cancer patients, informal caregivers and the general population

**DOI:** 10.1186/s12904-020-00594-1

**Published:** 2020-07-11

**Authors:** Mayara Goulart de Camargos, Bianca Sakamoto Ribeiro Paiva, Marco Antônio de Oliveira, Paula de Souza Ferreira, Vinicius Tolentino Nardoto de Almeida, Sandra de Andrade Cadamuro, Carla Simone Leite de Almeida, Carlos Eduardo Paiva

**Affiliations:** 1 Clinical Hospital of the Federal University of Triângulo Mineiro (UFTM), Uberaba, Minas Gerais Brazil; 2 Health-Related Quality of Life Research Group (GPQual), Learning and Research Institute, Barretos Cancer Hospital, Barretos, São Paulo, Brazil; 3 Center of Epidemiology and Biostatistics, Barretos Cancer Hospital, Barretos, São Paulo, Brazil; 4 Barretos School of Health Sciences, Dr. Paulo Prata – FACISB, Barretos, São Paulo, Brazil; 5 Federal Institute of Education, Science and Technology of Santa Catarina, Joinville, Santa Catarina Brazil; 6 Department of Clinical Oncology, Barretos Cancer Hospital, Barretos, São Paulo, Brazil

**Keywords:** Caregivers, Cancer, happiness, Subjective well-being, Health surveys, Patients, Personal satisfaction

## Abstract

**Background:**

Although cancer patients experience distressing symptoms and health-related changes in their quality of life, they may report positive emotional states. The lives of informal caregivers of cancer patients may also be affected by the patient’s cancer diagnosis; however, they may also find benefits in their experiences. Noticeable changes are reported in personal priorities after an oncologic diagnosis that can lead individuals to restructure their values and the way they perceive life. This study aims to assess happiness/satisfaction with life and positive and negative affect in cancer patients and informal caregivers compared with healthy people in the general population.

**Methods:**

A cross-sectional study with participants recruited online in five regions of Brazil through the social network site Facebook® and the application WhatsApp®. Surveys were completed using the SurveyMonkey® platform. A different sample of cancer patients and informal caregivers that was personally interviewed with the same forms was also grouped in the present analysis. Variables with *p*-values < 0.05 in the univariate analysis were included in linear regression models (stepwise, backward).

**Results:**

A total of 2580 participants were included, of whom 2112 were healthy representatives of the general population, 342 were cancer patients, and 126 were informal caregivers of cancer patients. In the multivariate analysis, the cancer patients and informal caregivers were happier than the healthy people in the general population, even after controlling for age, sex, educational level, and income. The patients and caregivers had lower scores for positive affect and higher scores for negative affect.

**Conclusions:**

Overall, the conditions related to happiness, satisfaction with life and positive affect are similar for all groups. However, cancer patients and informal caregivers report increased rates of happiness and satisfaction with life compared with theoretically healthy people, although they have lower positive affect scores and higher negative affect scores. It is suggested that cancer patients and caregivers of cancer patients experience more difficulties (suffering) on ​​a daily basis. However, given the increased difficulties, they perceive life differently, reporting that they are happier.

## Background

The definitions of happiness are diverse; however, they are most often related to a multidimensional construct or a positive emotional state with feelings of well-being. Happiness can be considered synonymous with subjective well-being and satisfaction with life [1, 2]. Happiness is an internal experience based on which each individual issues a judgment about his or her life and how and why he or she experiences it positively [2–4]. It is the consensus that regardless of how happiness is understood, human beings are always in search of it [4]. Happiness indexes are also used as indicators of economic growth and social development in several countries and influence the implementation of public policies [5]. Moreover, happiness is considered a basic human goal by the United Nations [6].

Cancer is one of the diseases most feared by society due to the suffering caused by its physical, emotional, social and spiritual effects. Although patients present with distressing symptoms associated with disease progression and even changes in health-related quality of life (HRQOL), they also report positive emotional states [2, 7, 8]. The lives of caregivers of cancer patients may be affected by the cancer diagnosis [9–11] because they help patients deal with functional, clinical, and psychosocial issues [12]. All of these factors can play a critical role in caregivers’ mental health and quality of life [13, 14]. However, caregivers may also find benefits in these experiences that may be associated with better outcomes in terms of well-being and happiness [15–17].

Considering that the cancer diagnosis is the beginning of an unknown journey, the path of this threatening experience can be marked by severe physical and emotional trauma [7, 8]. However, there are different ways of responding to the stressful nature of the cancer experience [18]. From a psychological point of view, cancer can be considered a psychosocial transition with potential for positive and negative changes [19]. Perceptible changes are reported in personal value priorities ​​after a cancer diagnosis, and can lead individuals to restructure their values and the way they perceive life [20, 21]. One question worthy of further study is the relation between current suffering and the perception of happiness/life satisfaction. Can individuals suffering from a life-threatening disease, such as cancer, be happy? On the other hand, does the absence of negative affects imply happiness? Given the suffering caused by cancer and the possibility of posttraumatic growth with possible change in the perception of life, the aim of this study was to evaluate the happiness/life satisfaction and the positive and negative affect of cancer patients and informal caregivers of cancer patients compared with healthy people in the general population.

With a focus on positive psychology, our main hypothesis was that cancer patients and informal caregivers of cancer patients should report levels of happiness and life satisfaction that are equal to or higher than those of healthy people despite reporting higher levels of negative affect. In addition, patients who have faced cancer and believe that they have overcome it (such as cancer survivors), as well as patients who are currently facing cancer and have a real chance of overcoming it (those undergoing adjuvant chemotherapy), should report higher levels of happiness and satisfaction with life than patients with no possibility of cure (those receiving palliative care only).

## Methods

### Study design

A pooled analysis of two cross-sectional samples: [1] healthy social media users who completed surveys using electronic tools and [2] cancer patients and informal caregivers of cancer patients who answered face-to-face questionnaires.

### Study participants

Individuals who have a Facebook® account and/or used the WhatsApp® application and caregivers of cancer patients and cancer patients treated at a cancer hospital.

### Study site

Participants from the five regions of Brazil were recruited online through the social network site Facebook® and the WhatsApp® application. In brief, three different strategies were used for the Facebook® survey: [1] From a total of 5570 Brazilian cities, 300 were randomly selected. One of the researchers (MCG) searched for Facebook® pages that could be representative of the city (preferentially official prefecture pages) and requested permission from the owners of those pages to post the link to the survey [2]; three university professors from the northeastern, northern and southern regions of Brazil were invited to post the research link on their personal Facebook® pages [3]; the authors posted the link on their personal pages. Additionally, people from the authors’ contact list networks were also contacted via the Whatsapp® application to disseminate and replicate the research so that the invitation to participate would reach as many Brazilian individuals as possible. In this case, explanatory text about the study was sent with the research link. In addition to being asked to respond to the questionnaire online (using either Facebook® or WhatsApp®), the participants were encouraged to share the study text/link with their own Facebook® and WhatsApp® contacts. The survey was completed using the SurveyMonkey® platform. Unfortunately, regardless of the participation route (Facebook or WhatsApp), all responses completed online were sent to the SurveyMonkey program data collector (the program through which the responses were collected and stored). Because the collector was the same for all methodologies, it was not possible to accurately identify the most effective search strategy.

A convenience sample of cancer patients and informal caregivers was interviewed in person using the same evaluation forms that were used for the online survey. Three trained interviewers (two research nurses and one medical student) collected all the data. Patients were recruited from oncology outpatient clinics, and caregivers were recruited from two different institutional support houses where cancer patients and informal caregivers from other locations stay while being treated in the city of Barretos.

### Eligibility criteria

Individuals who met all the inclusion criteria and none of the exclusion criteria were included.

#### General population participants

Inclusion criteria: Individuals of Brazilian nationality residing in the various Brazilian municipalities who had a Facebook® account and/or used the WhatsApp® application. Exclusion criteria: People under 18 years old.

#### Informal caregivers of cancer patients (people who were accompanying cancer patients at the time of the interview, whether or not they were relatives of the patient)

Inclusion criteria: Individuals of Brazilian nationality, aged ≥18 years, who accompanied a cancer patient during their treatment/follow-up at the cancer hospital and who could read and write. Exclusion criteria: Any relevant neuropsychiatric condition that would prevent the caregiver from understanding and answering health questionnaires (the researcher himself, before conducting the interview, assessed the conditions).

#### Cancer patients

Inclusion criteria: Individuals with a histological diagnosis of cancer of any histology or clinical stage, age between 18 and 75 years old, any sex, who could read and write and were in one of the following treatment stages: no evidence of disease and no cancer treatment for at least 2 years; receiving systemic adjuvant treatment; and under exclusive palliative care. Exclusion criteria: Any relevant neuropsychiatric condition that would prevent the patient from understanding and answering health questionnaires and the presence of hematological tumors. Neuropathological issues were identified through the evaluation of medical charts and as per the investigator’s evaluation. No cognitive impairment screening instrument was used. Hematological cancers were excluded because of logistical issues, since in the hospital, hematological cancer patients are treated in a different physical area outside the hospital.

### Sample size calculation

The sample size was calculated using the Power Analysis and Sample Size application (PASS v. 2002) via multiple linear regression analysis with seven predictors and a priori coefficients of determination (effect size) of *R*^2^ = 0.01, α = 0.05 and β = 0.05, thus resulting in an a priori statistical power of 95%. The estimated sample size was 2191 healthy participants.

The participants in this study (general population, cancer patients and informal caregivers) were grouped so that the sample size calculation for each group was performed separately. However, one yet-unpublished study separately examined patients and caregivers. The sample size calculated in that study aimed to compare happiness levels according to different subgroups of patients: [1] survivors (no evidence of disease and no antineoplastic treatment) [2]; patients receiving adjuvant therapy (those with no evidence of disease but receiving antineoplastic treatment); and [3] patients receiving palliative care only (those with evidence of disease and no antineoplastic treatment). Thus, assuming a rate of 70 and 50% of happy individuals in groups 1 and 3, α = 0.05, β = 0.10, at least 100 patients for each group should be included. Considering that caregivers would mainly be caring for patients whose profiles were similar to those of group 3, initial planning expected that caregivers and patients from group 3 would be compared. Thus, the minimum number of caregivers was also defined as 100.

The final sample comprised a minimum of 2591 participants.

### Study participant selection

Data were collected from October 2015 to October 2016. A total of 134 caregivers of cancer patients were invited to participate in the study. Eight refused to participate, and 126 were included. SurveyMonkey® (via Facebook and WhatsApp) collected data from 2688 respondents, but 90 were under 18 years old, and 13 declined, remaining 2585. Of these, 433 did not complete the Pemberton Happiness Index (PHI) and Satisfaction with Life Scale (SWLS), and 40 reported having cancer at the time of survey completion; thus, a total of 2112 responses were analyzed using the online tool. A total of 328 cancer patients were invited to participate in the study; 26 refused to participate, and the other 302 were included. Forty online respondents who reported having cancer at the time of survey completion were added to these, for a total of 342 cancer patients. The final sample included 2580 participants (2112 online respondents from the general population, 342 cancer patients and 126 caregivers).

### Ethical aspects

The study followed Resolution CNS 466/12, which regulates Brazilian ethical research issues. In Brazil, there are several accredited local Research Ethics Committees and a National Research Ethics Commission (CONEP). There are well-established criteria that define the need for additional approval by CONEP; the present study was approved by the Research Ethics Committee of the Barretos Cancer Hospital (opinion no. 1.098.789 and 1.114.730) and did not need to be sent to CONEP for further approval. All participants read and signed the informed consent form (ICF) before voluntarily agreeing to participate via either the online or the face-to-face formats.

### Data collection instruments

#### Questionnaire on sociodemographic and clinical characteristics and issues potentially associated with feeling of happiness

For the development of this questionnaire, the items were defined after meetings with researchers from the Palliative Care and Quality of Life Research Group (GPQual) and were based on a literature review and discussions about potential factors related to happiness. Before the main data collection process began, the responses of the first 50 individuals who completed the questionnaire were checked to assess accuracy, determine the frequency of missing items, and verify SurveyMonkey’s functioning. The questionnaire included sociodemographic data such as age, sex, marital status, religion, and demographic region, as well as clinical data, such as personal perception of health and previously diagnosed health problems, including psychiatric and/or psychological diagnoses. Several items addressed issues potentially associated with the perception of happiness (Supplementary Material 1).

#### Pemberton happiness index (PHI)

The PHI consists of eleven items related to different domains of remembered well-being (general, hedonic, eudaimonic and social) and ten items related to recent well-being (the previous day’s events). Responses were provided on a Likert scale, with higher indicating greater happiness. The sum of the items produces a combined well-being index (total PHI) [22]. The Portuguese version is valid and reliable for use in the Brazilian population through online surveys [23]. In the present study, the PHI-remembered (PHI-r) score was used. The Cronbach’s α was 0.877.

#### Satisfaction with life scale (SWLS)

The SWLS consists of five items that evaluate a cognitive component of life satisfaction, and participants answer on a seven-point scale ranging from 1 (totally disagree) to 7 (totally agree). In the Brazilian validation [24], the Cronbach’s α was 0.89. The SWLS is the most widely used scale for assessing overall life satisfaction and has been implemented in several languages ​​and cultures, thus providing a good psychometric index [25, 26]. In the present study, Cronbach’s α was 0.873.

#### Diener and Emmons’ positive and negative experience scale (PNES)

The PNES seeks to assess subjective well-being and the constructs of positive and negative affect. The original PNES is composed of nine items (four positive and five negative), which are answered on a 7-point Likert scale [3]. In a previous Brazilian study, the scale included 10 items, with the addition of the item “optimistic”, and revealed adequate psychometric parameters [27]. In the present study, Cronbach’s αs were 0.803 (positive affect) and 0.746 (negative affect).

### Statistical analysis

The sample was described using absolute and relative frequencies. Data from participants who answered at least the PHI and SWLS were included in the statistical analysis. The data normality was tested using the Kolmogorov-Smirnov test. Initially, the variables potentially related to the study outcomes were those associated with happiness (PHI), life satisfaction (SWLS), and negative/positive affect (PNES). Categorical variables with 2 and 3 categories were compared by means of the Mann-Whitney and Kruskal-Wallis tests, respectively. Only variables with *p* < 0.05 in the univariate analyses were included in the multiple linear regression models, which were adjusted for age, sex, income, and education. A significance level of 5% was adopted, and the analyses were performed in SPSS v.21.0.

Additional analyses were conducted to compare individual item scores on the PHI between groups of participants by means of the Kruskal-Wallis test. Scores for the items that evaluate “personal growth” and “meaning in life”, as well as an item that measures “the ability to enjoy small things in daily life”, were chosen. Additionally, an item measuring “bad moments in daily life” was chosen to validate the hypothesis of greater perceived happiness among those facing cancer (informal caregivers and cancer patients) despite reporting more suffering/negative affect in daily life. The main contents of and item descriptions for the selected PHI items were as follows: eudaimonic well-being/life meaning (“I think my life is useful and worthwhile”); eudaimonic well-being/personal growth (“My life is full of learning experiences and challenges that make me grow”); hedonic well-being/positive affect (I enjoy many little things every day); and hedonic well-being/negative affect (“I have many bad moments in my daily life”).

## Results

A total of 2580 participants were included in the study, of whom 2112 (81.9%) were from the general population, 342 (13.3%) were cancer patients, and 126 (4.9%) were informal caregivers of cancer patients. The majority were female (76.0%), white (67.0%), 18–29 years old (38.1%), married (49.1%), residing in the southeastern region of the country (47.6%) and living in an urban area (95.9%). Table 1 presents the sociodemographic characteristics of the study participants. **Supplementary Fig. 1** shows the flowchart of study participant selection.

**Table 1:** Socio-demographic characteristics of participants (*n* = 2580)

**Characteristics**	***Total (n* = 2580)**	**General Population (*n*** **= 2112)**	**Cancer Patients (*n*** **= 342)**	**Family caregivers (*n*** **= 126)**	***p*-value**
	**n (%)**	**n (%)**	**n (%)**	**n (%)**	
Gender					< 0.001
*Male*	620 (24.0)	479 (22.3)	122 (35.7)	28 (22.2)	
*Female*	1960 (76.0)	1672 (77.7)	220 (64.3)	98 (77.8)	
Race (ethnicity)					< 0.001
*White*	1729 (67.0)	1483 (70.2)	207 (60.5)	39 (31.0)	
*Black*	131 (5.1)	83 (3.9)	30 (8.8)	18 (14.3)	
*Latino*	633 (24.5)	476 (22.5)	90 (26.3)	67 (53.2)	
*Asian*	64 (2.5)	55 (2.6)	07 (2.0)	02 (1.6)	
*Missing*	23 (0.9)	15 (0.7)	08 (2.3)	00 (0.0)	
Age (years)					< 0.001
*18–29*	983 (38.1)	938 (44.4)	17 (5.0)	28 (22.2)	
*30–39*	765 (29.7)	699 (33.1)	24 (7.0)	42 (33.3)	
*40–49*	364 (14.1)	277 (13.1)	68 (19.9)	19 (15.1)	
*50–59*	292 (11.3)	141 (6.7)	124 (36.3)	27 (21.4)	
*60–69*	133 (5.2)	47 (2.2)	78 (22.8)	08 (6.3)	
*≥ 70*	42 (1.6)	10 (0.5)	30 (8.8)	02 (1.6)	
*Missing*	01 (0.0)	00 (00)	01 (0.3)	00 (0.0)	
Marital status					< 0.001
*Married*	1266 (49.1)	1002 (47.4)	189 (55.3)	75 (59.5)	
*Windowed*	64 (2.5)	24 (1.1)	35 (10.2)	05 (4.0)	
*Separated or divorced*	179 (6.9)	123 (5.8)	48 (14.0)	08 (6.3)	
*Single*	1053 (40.8)	951 (45.0)	64 (18.7)	38 (30.2)	
*Other / Do not Know*	18 (1.4)	12 (0.6)	06 (1.8)	00 (0.0)	
Place of residence (Brazilian region)					< 0.001
*North*	244 (9.5)	168 (8.0)	19 (5.6)	57 (45.2)	
*Northeast*	264 (10.2)	240 (11.4)	06 (1.8)	18 (14.3)	
*Southeast*	1228 (47.6)	964 (45.6)	264 (77.2)	00 (0.0)	
*Midwest*	273 (10.6)	179 (8.5)	46 (13.5)	48 (38.1)	
*South*	571 (22.1)	561 (26.6)	07 (2.0)	03 (2.4)	
Location where live					< 0.001
*Urban Area*	2473 (95.9)	2060 (97.5)	312 (91.2)	101 (80.2)	
*Rural Area*	107 (4.1)	52 (2.5)	30 (8.8)	25 (19.8)	
Educational level					< 0.001
*< 8 years of education*	259 (10.0)	26 (1.2)	186 (54.4)	47 (37.3)	
*8 to 11 years of education*	349 (13.5)	218 (10.3)	77 (22.5)	54 (42.9)	
*> 11 years of education*	1970 (76.4)	1866 (88.4)	79 (23.1)	25 (19.8)	
*Missing*	02 (0.1)	02 (0.1)	00 (0.0)	00 (0.0)	
Professional activity currently					< 0.001
*Yes*	2427 (94.1)	2030 (96.1)	282 (82.5)	115 (91.3)	
*No*	153 (5.9)	82 (3.9)	60 (17.5)	11 (8.7)	
Family income*					< 0.001
*≤ 3.9 minimum wages*	703 (27.2)	413 (19.6)	185 (54.1)	105 (83.3)	
*≥ 4 minimum wages*	1877 (72.8)	1699 (80.4)	157 (45.9)	21 (16.7)	
Religions beliefs					< 0.001
*Catholic*	1310 (50.8)	1058 (50.1)	197 (57.6)	55 (43.7)	
*Evangelic*	436 (16.9)	333 (15.8)	94 (27.5)	09 (7.1)	
*Spiritist*	413 (16.0)	382 (18.1)	30 (8.8)	01 (0.8)	
*Other*	28 (1.1)	24 (1.1)	02 (0.6)	02 (1.6)	
*No formal religion*	387 (15.0)	309 (14.6)	19 (5.6)	59 (46.8)	
*Atheist or agnostic*	06 (0.2)	06 (0.3)	00 (0.0)	00 (0.0)	

*Brazilian minimum wage

Univariate analyses were performed for each of the instruments used (PHI-r, SWLS, PNES). Statistically significant variables were included in adjusted multiple linear regression models. In total, 50 variables were tested; 58% (29/50) of the PHI-r items were considered significant, as well as 60% (30/50) of the SWLS items, 66% (33/50) of the positive affect items on the PNES and 54% (27/50) of the negative affect items on the PNES. The detailed results of the univariate analyses are presented in the supplemental materials (**Supplementary Materials 2 to 5**).

### PHI-r

In the multivariate model, the informal caregivers (β = 0.8, *p* ≤ 0.001) and cancer patients (β = 0.5, p ≤ 0.001) presented higher rates of happiness in relation to the general population. A positive self-assessment of health (β = 0.6, *p* ≤ 0.001) and reports of optimism (β = 1.4, p = < 0.001) were associated with increased happiness scores. Regarding aspects of the participants’ daily life, a higher frequency of family gatherings (β = 0.4, *p* ≤ 0.001), contact with nature (β = 0.2, *p* = 0.044), and leisure time (β = 0.3, p ≤ 0.001), as well as physical activity ≥3 times per week (β = 0.3, p ≤ 0.001), were associated with higher PHI scores in the multivariate model. In contrast, previous diagnoses of depression (β = − 0.7, *p* ≤ 0.001), anxiety (β = − 0.3, p ≤ 0.001) and other psychiatric/psychological problems (β = − 0.7, *p* = 0.002) were negatively associated with happiness scores (Table 2).

**Table 2:** Multiple Linear Regression for evaluation of happiness associated characteristics measured by PHI-r (n = 2580)

**Variables**	**β (SE)**	**IC95%**	***p*-Value**
Constant	4.4 (0.2)	4.1–4.8	< 0.001
Participants			
*General population*	–	–	–
*Caregivers of cancer patients*	0.8 (0.2)	0.4–1.1	< 0.001
*Cancer patients*	0.5 (0.1)	0.2–0.8	< 0.001
Feeling of happiness with the professional activity			
*Little*^*1*^	–	–	–
*Much*^*2*^	0.6 (0.1)	0.5–0.8	< 0.001
Self-assessment of health			
*Bad*^*3*^	–	–	–
*Good*^*4*^	0.6 (0.1)	0.4–0.8	< 0.001
Diagnosis of depression			
*No*	–	–	–
*Yes*	−0.7 (0.1)	−0.9 – (−0.4)	< 0.001
Diagnosis of anxiety			
*No*	–	–	–
*Yes*	−0.3 (0.1)	−0.5 – (− 0.1)	< 0.001
Other psychological/psychiatric problem			
*No*	–	–	–
*Yes*	−0.7 (0.2)	−1.2 – (− 0.3)	0.002
Current professional activity			
*Yes*	–	–	–
*No*	−0.3 (0.1)	−0.6 – (0.0)	0.028
Satisfaction with financial issues			
*Little*^*1*^	–	–	–
*Much*^*2*^	0.5 (0.1)	0.4–0.6	< 0.001
Frequency of family gatherings			
*Little*^*5*^	–	–	–
*Much*^*6*^	0.4 (0.1)	0.2–0.5	< 0.001
Influence of religious or spiritual life on happiness			
*Little*^*1*^	–	–	–
*Much*^*2*^	0.3 (0.1)	0.2–0.5	< 0.001
Self described as			
*Pessimistic*	–	–	–
*Neither optimistic nor pessimistic*	0.7 (0.1)	0.4–0.9	< 0.001
*Optimistic*	1.4 (0.1)	1.1–1.6	< 0.001
Contact with nature			
*Little*^*5*^	–	–	–
*Much*^*6*^	0.2 (0.1)	0.0–0.3	0.044
Leisure time			
*Little*^*1*^	–	–	–
*Much*^*2*^	0.3 (0.1)	0.2–0.5	< 0.001
Physical activity			
*Don’t practice physical activity*	–	–	–
*Once to twice per week*	0.0 (0.1)	−0.1 – 0.2	0.825
*3 or more times per week*	0.3 (0.1)	0.1–0.4	< 0.001

Model adjusted for the variables: age, gender, family income and education level (R^2^ = 0.598)

^1^*nothing/very little/more or less.*^*2*^*fairly/extremely.*^*3*^*very poor/poor/neither bad nor good.*^*4*^*good/very good.*^*5*^*nothing/very little/more or less.*^*6*^*many times/always*

### SWLS

Regarding life satisfaction, the informal caregivers (β = 2.9, *p* ≤ 0.001) and cancer patients (β = 0.9, *p* = 0.051) had higher scores than the general population. In the multivariate model, greater life satisfaction was associated with positive self-assessment of health (β = 2.3, *p* ≤ 0.001), optimism (β = 3.4, p ≤ 0.001), happiness with work (β = 2.4, *p* ≤ 0.001) and satisfaction with financial issues (β = 3.4, p ≤ 0.001). Being separated or divorced (β = − 2.0, *p* ≤ 0.001) and having a previous diagnosis of depression (β = − 2.5, *p* ≤ 0.001) or anxiety (β = − 0.9, *p* = 0.002) were negatively associated with life satisfaction (Table 3).

**Table 3:** Multiple Linear Regression for evaluation of characteristics associated with satisfaction with life, measured by SWLS (*n* = 2580)

**Variables**	**β (SE)**	**IC95%**	***p*-Value**
Constant	15.1(0.7)	13.8–16.4	< 0.001
Participants			
*General population*	–	–	–
*Caregivers of cancer patients*	2.9 (0.6)	1.7. – 4.2	< 0.001
*Cancer patients*	0.9 (0.5)	0.0–1.9	0.051
Marital status			
*Married*	–	–	–
*Windowed*	−1.1 (0.7)	−2.6 – 0.3	0.116
*Separated or divorced*	−2.0 (0.4)	− 2.9 (− 1.1)	< 0.001
*Single*	−0.2 (0.3)	−0.7 – 0.3	0.536
Feeling of happiness with the professional activity			
*Little*^*1*^	–	–	–
*Much*^*2*^	2.4 (0.2)	1.9–2.9	< 0.001
Self-assessment of health			
*Bad*^*3*^	–	–	–
*Good*^*4*^	2.3 (0.3)	1.7–2.9	< 0.001
Diagnosis of depression			
*No*	–	–	–
*Yes*	−2.5 (0.4)	−3.4 – (−1.7)	< 0.001
Diagnosis of anxiety			
*No*	–	–	–
*Yes*	−0.9 (0.3)	−1.5 – (−0.3)	0.002
Place of residence (Brazilian region)			
*Southeast*	–	–	–
*Midwest*	−0.9 (0.3)	−1.6 - (− 0.2)	0.016
*Northeast*	−0.7 (0.4)	−1.4 – 0.1	0.080
*North*	0.4 (0.4)	−0.4 – 1.2	0.303
*South*	0.1 (0.3)	−0.5 – 0.6	0.898
Satisfaction with financial issues			
*Little*^*1*^	–	–	–
*Much*^*2*^	3.4 (0.2)	2.9–3.9	< 0.001
Frequency of family gatherings			
*Little*^*5*^	–	–	–
*Much*^*6*^	1.3 (0.2)	0.9–1.8	< 0.001
Influence of religious or spiritual life on happiness			
*Little*^*1*^	–	–	–
*Much*^*2*^	1.1 (0.2)	0.6–1.6	< 0.001
Self described as			
*Pessimistic*	–	–	–
*Neither optimistic nor pessimistic*	1.5 (0.5)	0.6–2.5	0.002
*Optimistic*	3.4 (0.5)	2.5–4.4	< 0.001
Leisure time			
*Little*^*1*^	–	–	–
*Much*^*2*^	1.4 (0.2)	0.9–1.9	< 0.001
Physical activity			
*Don’t practice physical activity*	–	–	–
*Once to twice per week*	0.3 (0.3)	−0.2 – 0.9	0.230
*3 or more times per week*	0.8 (0.3)	0.2–1.3	0.004

Model adjusted for the variables: age, gender, family income and education level (R^2^ = 0.633)

^1^*nothing/very little/more or less.*^*2*^*fairly/extremely.*^*3*^*very poor/poor/neither bad nor good.*^*4*^*good/very good.*^*5*^*nothing/very little/more or less.*^*6*^*many times/always*

### PNES

Higher positive affect scores were associated reports of optimism (β = 5.2, *p* ≤ 0.001), positive self-assessments of health (β = 1.2, *p* = 0.001), happiness with work (β = 1.5, *p* ≤ 0.001), satisfaction with financial issues (β = 1.9, p ≤ 0.001) and leisure time (β = 1.7, *p* < 0.001). Lower positive affect levels were noted in cancer patients (β = − 3.8, p ≤ 0.001) and informal caregivers (β = − 2.0, *p* ≤ 0.001) than in the general population. Previous diagnoses of depression (β = − 2.1, *p* ≤ 0.001), anxiety (β = − 0.9, p ≤ 0.001), and some other psychiatric/psychological problems (β = − 1.4, *p* = 0.027) and not having a job (β = − 1.0, *p* = 0.007) were also associated with lower positive affect levels (Table 4).

**Table 4:** Multiple Linear Regression for evaluation of characteristics associated with positive affects measured by PNES (*n* = 2580)

**Variables**	**β (SE)**	**IC95%**	***p*-Value**
Constant	17.9(0.5)	16.9–18.9	< 0.001
Participants			
*General population*	–	–	–
*Caregivers of cancer patients*	−2.0 (0.5)	− 2.9 – (− 1.1)	< 0.001
*Cancer patients*	−3.8 (0.4)	−4.5 – (−3.0)	< 0.001
Current professional activity			
*Yes*	–	–	–
*No*	−1.0 (0.4)	− 1.7 – (− 0.3)	0.007
Voluntary activity			
*No*	–	–	–
*Yes*	0.3 (0.2)	0.0–0.7	0.092
Voluntary financial donation			
*No*	–	–	–
*Yes*	0.7 (0.2)	0.3–1.1	< 0.001
Influence of religious or spiritual life on happiness			
*Little*^*1*^	–	–	–
*Much*^*2*^	1.5 (0.2)	1.2–1.9	< 0.001
Self-assessment of health			
*Bad*^*3*^	–	–	–
*Good*^*4*^	1.2 (0.2)	0.7–1.6	0.001
Diagnosis of depression			
*No*	–	–	–
*Yes*	−2.1 (0.3)	−2.8 – (−1.5)	< 0.001
Diagnosis of anxiety			
*No*	–	–	–
*Yes*	−0.9 (0.2)	−1.4 – (−0.4)	< 0.001
Other psychological/psychiatric problem			
*No*	–	–	–
*Yes*	−1.4 (0.6)	−2.6 – (−0.2)	0.027
Frequency of family gatherings			
*Little*^*5*^	–	–	–
*Much*^*6*^	0.7 (0.2)	0.3–1.0	< 0.001
Influence of religious or spiritual life on happiness			
*Little*^*1*^	–	–	–
*Much*^*2*^	0.5 (0.2)	0.1–0.9	0.006
Satisfaction with financial issues			
*Little*^*1*^	–	–	–
*Much*^*2*^	1.9 (0.2)	1.5–2.2	< 0.001
Self described as			
*Pessimistic*	–	–	–
*Neither optimistic nor pessimistic*	2.1 (0.4)	1.3–2.9	< 0.001
*Optimistic*	5.2 (0.4)	4.5–6.0	< 0.001
Leisure time			
*Little*^*1*^	–	–	–
*Much*^*2*^	1.7 (0.2)	1.3–2.1	< 0.001
Physical activity			
*Don’t practice physical activity*	–	–	–
*Once to twice per week*	0.2 (0.2)	−0.3 – 0.6	0.487
*3 or more times per week*	0.5 (0.2)	0.1–0.9	0.010

Model adjusted for the variables: age, gender, family income and education level (R^2^ = 0.651)

^1^*nothing/very little/more or less.*^*2*^*fairly/extremely.*^*3*^*very poor/poor/neither bad nor good.*^*4*^*good/very good.*^*5*^*nothing/very little/more or less.*^*6*^*many times/always*

Subsequently, higher levels of negative affect were observed in the informal caregivers (β = 3.6, *p* ≤ 0.001) and cancer patients (β = 2.3, p ≤ 0.001) than in the general population. Individuals with a previous diagnosis of depression (β = 2.2, p ≤ 0.001) and anxiety (β = 1.5, p ≤ 0.001) also exhibited higher negative affect levels. In contrast, a positive self-assessment of health (β = − 0.7, *p* = 0.005), optimism (β = − 3.5, p ≤ 0.001), happiness with work (β = − 1.3, p ≤ 0.001), satisfaction with financial issues (β = − 0.9, p ≤ 0.001) and not having a sick loved one (β = − 0.5, p = 0.005) were associated with lower negative affect levels (Table 5).

**Table 5:** Multiple Linear Regression for evaluation of characteristics associated with negative affects measured by PNES (*n* = 2580)

**Variables**	**β (SE)**	**IC95%**	***p*-Value**
Constant	17.7(0.5)	16.7–18.8	< 0.001
Participants			
*General population*	–	–	–
*Caregivers of cancer patients*	3.6 (0.5)	2.7–4.5	< 0.001
*Cancer patients*	2.3 (0.4)	1.6–3.0	< 0.001
Feeling of happiness with the professional activity			
*Little*^*1*^	–	–	–
*Much*^*2*^	− 1.3 (0.2)	−1.6 – (− 0.9)	< 0.001
Self-assessment of health			
*Bad*^*3*^	–	–	–
*Good*^*4*^	−0.7 (0.2)	−1.1 – (− 0.2)	0.005
Sickness in a close person (a loved one)			
*Yes*	–	–	–
*No*	−0.5 (0.2)	−0.8 – (− 0.1)	0.005
Diagnosis of depression			
*No*	–	–	–
*Yes*	2.2 (0.3)	1.5–2.8	< 0.001
Diagnosis of anxiety			
*No*	–	–	–
*Yes*	1.5 (0.2)	1.0–1.9	< 0.001
Satisfaction with financial issues			
*Little*^*1*^	–	–	–
*Much*^*2*^	−0.9 (0.2)	−1.3 – (− 0.5)	< 0.001
Self described as			
*Pessimistic*	–	–	–
*Neither optimistic nor pessimistic*	−1.7 (0.4)	−2.5 – (−0.9)	< 0.001
*Optimistic*	−3.5 (0.4)	−4.2 – (− 2.7)	< 0.001
Leisure time			
*Little*^*1*^	–	–	–
*Much*^*2*^	−0.9 (0.2)	−1.3 – (− 0.5)	< 0.001
Physical activity			
*Don’t practice physical activity*	–	–	–
*Once to twice per week*	0.0 (0.2)	−0.4 – 0.5	0.901
*3 or more times per week*	−0.6 (0.2)	−0.9 – (− 0.1)	0.006

Model adjusted for the variables: age, gender, family income and education level (R^2^ = 0.544)

^1^*nothing/very little/more or less.*^*2*^*fairly/extremely.*^*3*^*very poor/poor/neither bad nor good.*^*4*^*good/very good.*^*5*^*nothing/very little/more or less.*^*6*^*many times/always*

#### Analyses among patient groups

**Fig. 1:**
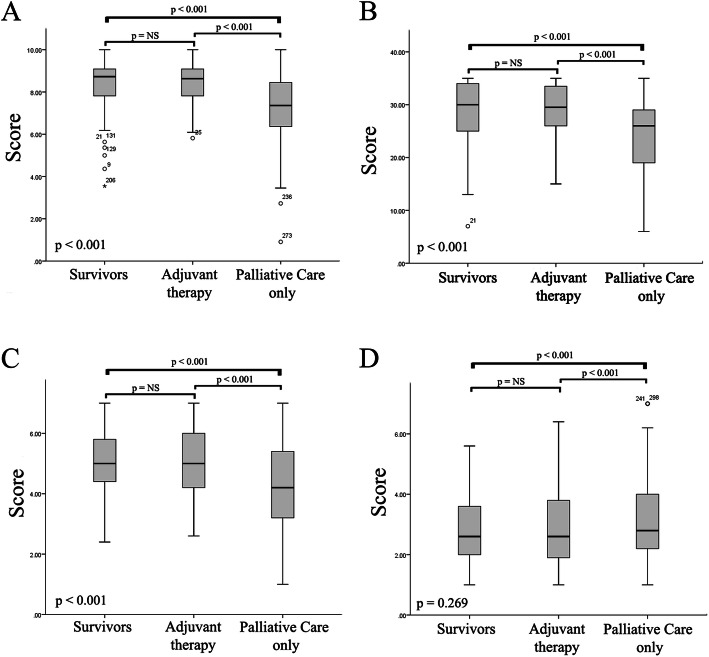
Comparisons of the PHI-r, SWLS, and PNES scores between patients with cancer at different phases of disease. The PHI-r (**a**), SWLS (**b**), PNES-positive (**c**), and PNES-negative (**d**) median scores were compared among three groups of patients: survivors (those with no evidence of disease and no antineoplastic treatment); adjuvant therapy (those with no evidence of disease but who were receiving antineoplastic treatment); and palliative care only (those with evidence of disease and not receiving antineoplastic treatment)

To better understand whether the results identified in the regression analyses applied to all patients, instrument scores were compared among three groups of patients: survivors, those receiving adjuvant therapy, and those receiving palliative care only. Figure 1 shows that the scores were similar between survivors and patients receiving adjuvant therapies; however, palliative care-only patients had lower PHI-r, SWLS and positive affect scores. No significant differences between the medians for negative affect were noted among the three groups. The level of significance (Bonferroni) was corrected and was *p* < 0.01667 (0.05/3) for multiple comparisons.

### PHI individual scores

The median and interquartile range (IQR) values of the PHI items were compared among the cancer patients, informal caregivers and healthy people: the “life meaning” item (cancer patients = 10(1); caregivers = 10(1); general population = 9(3); *p* < 0.001); the “personal growth” item (cancer patients = 10(2); caregivers = 10(2); general population = 9(4); *p* < 0.001); the “enjoy little things” item (cancer patients = 10(2); caregivers = 9(3); general population = 7(4); *p* < 0.001); and the “bad moments” item (cancer patients = 5(6); caregivers = 6(5); general population = 3(5); *p* < 0.001).

## Discussion

In this study, the perceptions of happiness, life satisfaction and positive and negative affect of a population of cancer patients and informal caregivers of cancer patients were measured and compared with the perceptions of healthy individuals from the general population who were social network users. Linear regression analyses confirmed the association of several personal, health, financial and work characteristics, leisure activities and rest with greater perceptions of happiness, life satisfaction and positive affect. Even after adjusting for age, sex, income and education, the cancer patients and informal caregivers were happier than the healthy people from the general population. Interestingly, the patients and caregivers presented lower positive affect scores and higher negative affect scores. The results prompted us to formulate the concept that despite experiencing more problems in daily life (which generate greater negative affect), individuals with cancer or with a loved one with cancer feel happier and more satisfied with life, likely due to changed expectations about the future and increased valuing of simpler aspects of everyday life.

### Main findings

The feeling of happiness is subject to a wide range of external influences; however, hereditary components are present that can be explained by the genetic architecture of the personality and are responsible for 30 to 50% of the score variance [4, 28, 29]. The “genetics of happiness” and personality traits maintain relatively constant happiness scores over time. However, engaging in activities that promote increased happiness may also potentially improve positive feeling levels for significant periods of time [4]. In this study, the final linear regression models explained between 54 and 65% of the variance in the participants’ scores for happiness, life satisfaction and positive and negative affect, suggesting that the remaining percentage of variance may be explained by genetic or personality-specific aspects that were not investigated.

In the present study, the characteristics associated with higher happiness scores enhance the multidimensional nature of the “happiness” construct. Some categories of factors that tend to positively influence the general population’s sense of happiness have been described in prior studies; these factors include optimism, the pursuit of goals and purposes, acts of kindness, expressions of gratitude, financial satisfaction, physical exercise, social relationships, rest, and altruism [30–32]. The present study was the first to detail happiness-related conditions in a large sample of the Brazilian population using properly validated tools.

For an adequate understanding of the results of this study, it should be emphasized that the scales used tend to measure slightly different constructs. The PNES uses more direct questions and investigates more transient life aspects. The SWLS and PHI-r include more reflective questions addressing existential aspects. Examples of such items are as follows: from the SWLS, “As much as possible, I have achieved the important things I want out of life”; from the PHI, “My life is full of learning experiences and challenges that make me grow,” and “I enjoy many little things every day”. These differences explain why patients and informal caregivers have lower positive and higher negative affect and simultaneously have better happiness and life satisfaction scores, which at first glance would appear incoherent. To validate this finding, a different analysis was performed; it determined that the scores for items that evaluate “personal growth” and “meaning in life” and for the item that measures “ability to enjoy small things in daily life” were higher among the patients and caregivers than among the healthy people. Again, both the patients and the caregivers reported higher scores on the “bad moments in daily life” item than the healthy people did.

Patients with cancer and cancer survivors who are happy tend to have a better quality of life and fewer symptoms than unhappy patients do. In addition, happier cancer patients are more likely to report positive expectations about the future; have more life goals; have greater optimism and hope; experience and positive changes within their relationships; are better at coping with problems; and have higher spirituality levels [18, 21, 33]. Acceptance of the cancer diagnosis and the “path” through treatment are often seen as psychosocial and spiritual transitions that can lead to a restructuring of values and can reflect how patients assess life and their sense of happiness [20, 21]. Thus, it is important to evaluate happiness in cancer patients and to seek methods to promote it, i.e., through providing support during treatment or encouraging the development of positive characteristics unrelated to the disease or its treatment. The role of the health professional should not be limited exclusively to issues related to the disease itself.

The psycho-oncological literature shows characteristics of posttraumatic growth in patients during and after treatment for cancer. This process refers to the positive psychological changes experienced as a result of struggling with highly challenging life circumstances [18]. The changes occur mainly in interpersonal relationships, perceptions of personal strength, appreciation of life, spirituality and values ​​priorities [18]. In the present study, it was possible to identify some aspects that reflect the posttraumatic growth of the participants who had cancer and the informal caregivers of patients with cancer. These groups even had higher happiness scores than the healthy people in the general population did.

The well-being of informal caregivers of cancer patients is also affected by the cancer diagnosis [10, 11]. Caring for suffering patients can cause a significant negative emotional load for their caregivers in addition to causing fatigue, physical exhaustion, etc. Despite these negative parameters, caregivers of cancer patients may present high well-being levels that are influenced by characteristics and events inherent to their own life and the lives of those for whom they care [17].

According to a Buddhist aphorism, “we all want to be happy and not suffer”. However, the results of this study suggest that suffering (either experiencing it oneself and/or observing it in someone close), although undesirable, has a role in individual growth in healthy individuals and individuals with chronic diseases (such as cancer) [15, 17, 20, 21]. Cancer patients exclusively receiving palliative care had reduced perceptions of happiness and life satisfaction compared with the other groups of cancer patients, as expected. In these patients, physical (pain, fatigue, nausea, dyspnea, among others) and psychosocial suffering are high, especially at the end of life. After adequate control of physical and psychological symptoms, strategies that focus on problem solving, personal issues, forgiveness and dignity therapy may play a fundamental role in the existential domain. If managed properly, these patients will probably report higher happiness and life satisfaction scores. However, this hypothesis needs to be tested in future studies.

A comparative study [34] of students from Brazil and Norway evaluated the use and efficiency of 14 strategies for regulating emotion with the aim of stopping anger, anxiety and sadness. Norway and Brazil were chosen because they were thought to represent individualistic and collectivistic cultures, respectively. In general, the Norwegians used a greater number of strategies than the Brazilians. Cultural differences were suggested by comparatively high user ratings of “working”, “acting out”, and “going for a walk” by Norwegians and “relaxation” and “entertainment” by Brazilians. The “pray to God” strategy was also more frequently used by Brazilians than by Norwegians. Thus, it is suggested that people from collectivist countries (such as Brazil) seek strategies for regulating emotions that foster individualism and self-knowledge. In the present study, at the time of emotional crisis (cancer diagnosis), the Brazilian participants presented higher scores for happiness and satisfaction with life, which may suggest that the pursuit of self-knowledge and individualization could have led to personal growth. However, subsequent studies need to be designed to explain these findings.

### Strengths and weaknesses

This study has some limitations. For example, the participating patients’ caregivers were not evaluated/included, and there was no information about the conditions of the patients whom the participating caregivers were accompanying; however, it was assumed that they were patients with reduced functionality given that many patients do not need caregivers when they are in good physical condition. Although the healthy participants were selected from the general population via social media, they probably do not characterize and represent the entire Brazilian population.

In contrast, the study is original because no other study has been conducted in Brazil that evaluates the constructs studied in the different groups addressed and because happiness is rarely measured in cancer patients. In addition, the study allowed the comparison of a large population of the same nationality and from different regions.

The pooled analysis of the general population and people affected by cancer, whether as patients or caregivers, has its limitations. Although statistical adjustment techniques were used, the combined populations have different profiles, which can be considered a potential selection bias. Our sample is consistent with the most recent Brazilian population census, which showed that the most populous region is the Southeast and that the largest number of white Brazilians live in urban areas and are Catholics; on the other hand, our study included a higher percentage of women and had a higher income and educational level than the general population described in the census. The modes of administration (on-line and face-to-face) also represent a study limitation. However, the authors were careful to use the same research instruments for data collection in both modes and to properly train the interviewers to standardize data collection. In addition, previous comparative studies [35, 36] have shown small or no magnitude of influence on the responses to the questionnaires.

## Conclusions

Cancer patients and informal caregivers of cancer patients reported higher levels of happiness and life satisfaction than healthy people despite having lower positive affect scores and higher negative affect scores. Further studies are needed to investigate the influence of posttraumatic growth on happiness and life satisfaction in patients with cancer and their caregivers

## Supplementary information

**Additional file 1: Supplementary Material 1.** Questionnaire on sociodemographic and clinical characteristics and issues potentially associated with feelings of happiness. Questionnaire used for the collection of data regarding sociodemographic and clinical characteristics and issues potentially associated with feelings of happiness.

**Additional file 2: Supplementary Material 2**. Univariate analysis for the evaluation of characteristics associated with happiness measured by the Pemberton Happiness Index (PHI-r) (*n* = 2580). Items used in the univariate analysis to assess the characteristics associated with happiness.

**Additional file 3: Supplementary Material 3**. Univariate analysis for the evaluation of characteristics associated with satisfaction with life measured by the Satisfaction with Life Scale (SWLS) (*n* = 2580). Items used in the univariate analysis to assess the characteristics associated with satisfaction with life.

**Additional file 4: Supplementary Material 4.** Univariate analysis for the evaluation of characteristics associated with positive affect measured by Diener and Emmon’s Positive and Negative Experience Scale (PNES) (n = 2580). Items used in the univariate analysis to assess the characteristics associated with positive affect.

**Additional file 5: Supplementary Material 5.** Univariate analysis for the evaluation of characteristics associated with negative affect measured by Diener and Emmon’s Positive and Negative Experience Scale (PNES) (n = 2580). Items used in the univariate analysis to assess the characteristics associated with negative affect.

## Data Availability

The datasets used and/or analyzed during the current study are available from the corresponding author on reasonable request. We have full control of all primary data, and we agree to allow the journal to review the data if requested.

## Abbreviations

GPQual: Palliative Care and Quality of Life Research Group
HRQOL: Health-related quality of life
ICGF: Informed consent form
PHI: Pemberton Happiness Index
PHI-r: PHI-remembered
PNES: Diener and Emmons’ Positive and Negative Experience Scale
SWLS: Satisfaction with Life Scale
