# Supplementary material for: An explorative analysis of the differences in levels of happiness between cancer patients, informal caregivers and the general population
Source: BMC Palliat Care. 2020 Jul 11;19:106. doi: 10.1186/s12904-020-00594-1 (PMC7354680; doi:10.1186/s12904-020-00594-1)
Supplement: Supplementary file 1 — Additional file 1: Supplementary Material 1. Questionnaire on sociodemographic and clinical characteristics and issues potentially associated with feelings of happiness. Questionnaire used for the collection of data regarding sociodemographic and clinical characteristics and issues potentially associated with feelings of happiness. [file 12904_2020_594_MOESM1_ESM.docx]

**Supplementary Material 1**

| **Questionnaire of socio-demographic, clinical characterization and questions potentially associated to the sensation of happiness** | | | | |
| --- | --- | --- | --- | --- |
| **Data completed by the researcher** | | | | |
| **1** | Study ID | | **1** |  |
| **2** | Initials | | **2** |  |
| **3** | Date of data collection  **MM/DD/AAAA** | | **3** |  |
| **4** | Sex  **1**- Female **2**- Male | | **4** |  |
| **5** | What race (ethnicity) do you consider yourself?  **1**-White **2**- Black **3**- Latino **4**- Asian **5**- Other _____________________ | | **5** |  |
| **6** | How old are you (in years)?  **1-** <18 **2**- 18-29 **3**- 30-39 **4**- 40-49 **5**- 50-59  **6**- 60-69  **7**- 70-79  **8**- ≥80 | | **6** |  |
| **7** | What is your current marital status?   1. Married or live married **2-** Widowed **3-** Separated or divorced **4-** Single   **5-** Do not know answer **6-** Other____________________________________________ | | **7** |  |
| **8** | What is your educational level (even when you studied)?  **1-** I never studied **2-** I never studied, but I can read and write **3-** I stopped studying before the fourth grade **4-** I completed the fourth grade **5-** I stopped studying before the eighth grade **6-** I completed the eighth grade **7-** I stopped studying before the third year **8-** I completed the third year **9-** I started, but I did not finish a college **10-** I finished college **11-** I did Post-Graduation **12-** Other ______________________________________ | | **8** |  |
| **09** | Do you feel happy with your professional activity? | | **09** |  |
|  | **1**- Nothing **2**- Very little **3**- More or less **4**- Fairly **5**- Extremely | |  |  |
| **10** | What state do you live in?  **1-** Acre (AC) **2-** Alagoas (AL) **3-** Amapá (AP) **4-** Amazonas (AM) **5-** Bahia (BA)  **6-**Ceará (CE) **7-** Distrito Federal (DF) **8-** Espírito Santo (ES) **9-** Goiás (GO)  **10-** Maranhão (MA) **11-** Mato Grosso (MT) **12-** Mato Grosso do Sul (MS) **13-** Minas Gerais (MG)  **14-** Pará (PA) **15-** Paraíba (PB) **16-** Paraná (PR) **17-** Pernambuco (PE)  **18-** Piauí (PI) **19-** Rio de Janeiro (RJ) **20-** Rio Grande do Norte (RN) **21-** Rio Grande do Sul (RS) **22-** Rondônia (RO) **23-** Roraima (RR) **24-** Santa Catarina (SC) **25-** São Paulo (SP) **26-** Sergipe (SE) **27-** Tocantins (TO) | | **10** |  |
| **11** | Is the place you live in an urban or rural area?  **1**- Urban **2**- Rural | | **11** |  |
| **12** | What is the total income of your household (including some kind of government aid)?  **1-** Less than de R$788,00  **2-** From R$789,00 to R$1575,00  **3-** From R$1576,00 to R$2363,00  **4-** From R$2364,00 to R$3939,00  **5-** From R$3940,00 to R$7879,00  **6-** From R$7880,00 to R$15.759,00  **7-**  More than R$15.760,00 | | **12** |  |
| **13** | Do you get some kind of government grant or scholarship? (you can check more than one answer) | | **13** |  |
|  | **0**- I don’t receive any  **1**- PROUNI (University Program for All)  **2**- FIES (Student Funding Program)  **3**- Citizen Funding Program  **4**- Work Funding Program  **5**- Food Funding Program  **6**- School Funding Program  **7**- Family Funding Program  **8**- Olympic Pouch  **9**- Cup Funding Program  **10**- Permanence Funding Program | **11**- Dictatorship grant  **12**- Pregnant women grant  **13**- Retirement for contribution time  **14**- Retirement by age  **15**- Retirement due to disability  **16**- Sickness Funding Program  **17**- Maternity Salary  **18**- Confinement aid  **19**- Pension by death  **20**- Other |  |  |
| **14** | How satisfied are you with what you have achieved financially in your life to this day?  **1-** Nothing satisfied **2-** Very unsatisfied **3-** More or less satisfied **4-** Very satisfied  **5-** Extremely satisfied | | **14** |  |
| **15** | How often do you and your family meet for a family gatherings at home (for example, lunch and family dinner)?   1. I don’t have family **1-** Never **2-** Rarely **3-** Sometimes **4-** Often **5-** Always | | **15** |  |
| **16** | Do you have any religion?  **0-** I don’t have **1-** Catholic **2-** Evangelical **3-** Spiritist **4-** Other_________________ | | **16** |  |
| **17** | To what extent does your religious or spiritual life influence your happiness? | | **17** |  |
|  | **1**- Nothing **2**- Very little **3**- More or less **4**- Fairly **5**- Extremely | |  |  |
| **18** | Have you done any of the activities below?  **1-** Volunteer work to help people or institutions  **2-** Financial donation to help people or institutions  **3-** I have not done any of these activities | | **18** |  |
| **19** | Do you have pet (can you mark more than one answer if necessary)?  **1-** No **2-** Yes, cat **3-** Yes, dog **4-** Yes, birds **5-** Yes, rabbit **6-** Yes, turtle  **7-** Yes, hamster/rabbit/rat **8-** Yes, fish **9-** Yes, other (specify)_________ | | **19** |  |
| **20** | How often do you have the opportunity to stay part of your day in the middle of nature (hiking in parks, rural areas, waterfalls, trails in forests, etc.) | | **20** |  |
|  | **1**- Never **2**- Rarely **3**- Sometimes **4-** Often **5-** Constantly | |  |  |
| **21** | You consider yourself a person:  **1**- Pessimistic **2**- Neither optimistic nor pessimistic **3**- Optimistic | | **21** |  |
| **22** | Considering the people you care about (close friends and family), is anyone currently sick?  **1-**Yes **2-** No | | **22** |  |
| **23** | How much of your happiness has been affected by the illness of the person you care about (close friend or relative)?  **1-** Nothing **2-** Very Little  **3-** More or Less **4-** Fairly **5-** Extremely **99-** Not Applicable | | **23** |  |
| **24** | How often have you been performing some kind of physical activity (walking, cycling, soccer, swimming, some kind of fighting, etc.)? | | **24** |  |
|  | **0**- I don’t perform physical activity **1**- 1 time per week **2**- 2 times per week **3**-3 times per week **4-** 4 times per week **5-** 5 times per week **6-** 6 times per week **7-** 7 times per week | |  |  |
| **24** | How far have you had leisure time (going out for walks, resting time, talking, traveling, theater, movie theater, etc.)? | | **24** |  |
|  | 1. Nothing **2-** Very Little **3-** More or Less **4-** Fairly  **5-** Extremely | |  |  |
| **25** | How do you consider your health?  **1-** Very poor **2-** Poor **3-** Neither bad nor good **4-** Good **5-** Very good | | **25** |  |
| **26** | Among the health problems below, select the one you are carrying (for which you take some medicine or have a diagnosis made by a doctor):  **1-** I do not currently have and never had an important health problem **2-** Cancer (CURRENTLY IN TREATMENT) **3-** Cancer (DIAGNOSED AND WITH TERMINATION OF TREATMENT LESS THAN 5 YEARS AGO) **4-** Cancer (DIAGNOSED AND WITH TERMINATION OF TREATMENT THERE IS MORE THAN 5 YEARS) **5-** Cirrhosis of the liver **6-** Hypertension (High blood pressure) **7-** Coronary problems **8-** Diabetes with complications **9-** Diabetes without complications **10-** Chronic lung problems (COPD, Pulmonary emphysema) **11-** Heart failure **12-** Renal insufficiency with dialysis  **13-** Renal insufficiency without dialysis **14-** Stroke sequel **15-** Depression **16-** Anxiety **17-** Panic Syndrome **18-** Another psychiatric or psychological problem **19-** Another health problem__________________________________________ | | **26** |  |
