# Supplementary material for: An explorative analysis of the differences in levels of happiness between cancer patients, informal caregivers and the general population
Source: BMC Palliat Care. 2020 Jul 11;19:106. doi: 10.1186/s12904-020-00594-1 (PMC7354680; doi:10.1186/s12904-020-00594-1)
Supplement: Supplementary file 2 — Additional file 2: Supplementary Material 2. Univariate analysis for the evaluation of characteristics associated with happiness measured by the Pemberton Happiness Index (PHI-r) (n = 2580). Items used in the univariate analysis to assess the characteristics associated with happiness. [file 12904_2020_594_MOESM2_ESM.docx]

| **Supplementary Material 2 –** Univariate analysis for the evaluation of characteristics associated with happiness measured by Pemberton Happiness Index (PHI-r) (n=2580). | | |
| --- | --- | --- |
| **Variables** | **Median (P25 – P75)** | **p-Value** |
| Participants |  | <0.001 |
| *General population* | 7.55 (5.86 - 8.45) |  |
| *Caregivers of cancer patients* | 8.27 (7.45 - 8.82) |  |
| *Cancer patients* | 8.27 (7.36 - 9.00) |  |
| Age (years) |  | <0.001 |
| *18-29* | 7.27 (5.73 - 8.27) |  |
| *30-39* | 7.64 (6.09 - 8.45) |  |
| *40-49* | 7.91 (6.55 - 8.73) |  |
| *50-59* | 8.18 (7.18 - 9.09) |  |
| *60-69* | 8.27 (7.27 - 8.82) |  |
| *≥70* | 8.45 (7.55 - 8.91) |  |
| Marital Status |  | <0.001 |
| *Married* | 7.91 (6.45 - 8.73) |  |
| *Windowed* | 8.23 (7.05 - 9.00) |  |
| *Separated or divorced* | 7.73 (6.45 - 8.64) |  |
| *Single* | 7.36 (5.91 - 8.36) |  |
| Educational Level |  | <0.001 |
| *<8 years of education* | 8.36 (7.36 – 9.00) |  |
| *8 to 11 years of education* | 7.82 (6.55 - 8.73) |  |
| *>11 years of education* | 7.55 (5.91 - 8.45) |  |
| Feeling of happiness with the professional activity |  | <0.001 |
| *Hasn’t professional activity* | 6.91 (5.00 - 8.27) |  |
| *Has professional activity* | 7.73 (6.27 - 8.64) |  |
| Location where live |  | <0.001 |
| *Urban Area* | 7.64 (6.09 - 8.55) |  |
| *Rural Area* | 8.27 (6.91 - 9.00) |  |
| Government aid |  | 0.001 |
| *Yes* | 7.82 (6.55 - 8.73) |  |
| *No* | 7.64 (6.09 - 8.55) |  |
| Disability retirement |  | 0.012 |
| *No* | 7.64 (6.18 - 8.55) |  |
| *Yes* | 8.18 (6.82 - 8.91) |  |
| Sickness aid |  | 0.012 |
| *No* | 7.64 (6.09 - 8.55) |  |
| *Yes* | 7.91 (6.82 - 8.86) |  |
| Religious beliefs |  | 0.003 |
| *Catholic* | 7.73 (6.27 - 8.64) |  |
| *Evangelic* | 7.64 (6.45 - 8.55) |  |
| *Spiritist* | 7.73 (6.00 - 8.64) |  |
| *Other* | 7.41 (5.00 - 8.36) |  |
| *Atheist / Agnostic / No formal religion* | 7.36 (5.64 - 8.36) |  |
| Voluntary activity |  | <0.001 |
| *No* | 7.55 (6.09 - 8.55) |  |
| *Yes* | 8.00 (6.64 - 8.73) |  |
| Voluntary financial donation |  | <0.001 |
| *No* | 7.45 (5.82 - 8.45) |  |
| *Yes* | 8.00 (6.82 - 8.82) |  |
| Cat as a pet |  | 0.009 |
| *No* | 7.73 (6.27 - 8.64) |  |
| *Yes* | 7.36 (5.45 - 8.45) |  |
| Self described as |  | <0.001 |
| *Pessimistic* | 5.55 (3.91 - 6.64) |  |
| *Neither optimistic nor pessimistic* | 6.82 (5.18 - 7.82) |  |
| *Optimistic* | 8.18 (7.09 - 8.82) |  |
| Current health problem |  | 0.002 |
| *Yes* | 7.55 (5.91 - 8.55) |  |
| *No* | 7.82 (6.36 - 8.64) |  |
| Diagnosis and treatment of current cancer |  | <0.001 |
| *No* | 7.55 (6.00 - 8.45) |  |
| *Yes* | 8.27 (7.36 - 9.00) |  |
| Diagnosis of depression |  | <0.001 |
| *No* | 7.82 (6.45 - 8.64) |  |
| *Yes* | 5.91 (4.00 - 7.09) |  |
| Diagnosis of anxiety |  | <0.001 |
| *No* | 7.91 (6.64 - 8.73) |  |
| *Yes* | 6.55 (4.82 - 7.91) |  |
| Diagnosis of panic disorder |  | <0.001 |
| *No* | 7.73 (6.27 - 8.64) |  |
| *Yes* | 5.45 (4.00 - 7.00) |  |
| Other psychological/psychiatric problem |  | <0.001 |
| *No* | 7.73 (6.27 - 8.64) |  |
| *Yes* | 5.55 (3.32 - 7.64) |  |
| Influence of religious or spiritual life on happiness |  | <0.001 |
| *Little¹* | 7.00 (5.23 - 8.18) |  |
| *Much²* | 8.00 (6.73 - 8.73) |  |
| Self-assessment of health |  | <0.001 |
| *Bad³* | 6.82 (4.82 - 8.09) |  |
| *Good* *^4^* | 7.82 (6.45 - 8.73) |  |
| Frequency of family gatherings |  | <0.001 |
| *Little ^5^* | 7.18 (5.45 - 8.18) |  |
| *Much^6^* | 8.00 (6.73 - 8.82) |  |
| Contact with nature |  | <0.001 |
| *Little ^5^* | 7.45 (5.91 - 8.45) |  |
| *Much^6^* | 8.27 (7.27 - 8.91) |  |
| Physical activity |  | <0.001 |
| *Don’t practice physical activity* | 7.55 (5.82 - 8.45) |  |
| *Once to twice per week* | 7.36 (6.09 - 8.36) |  |
| *3 or more times per week* | 8.09 (6.82 - 8.73) |  |
| Leisure time |  | <0.001 |
| *Little¹* | 7.36 (5.82 - 8.36) |  |
| *Much²* | 8.27 (7.09 - 8.91) |  |
| Feeling of happiness with the professional activity |  | <0.001 |
| *Little¹* | 6.82 (5.18 - 8.09) |  |
| *Much²* | 8.09 (7.00 - 8.82) |  |
| Satisfaction with financial issues |  | <0.001 |
| *Little¹* | 7.18 (5.55 - 8.18) |  |
| *Much²* | 8.27 (7.45 - 9.00) |  |
| Happiness affected by loved one's disease |  | 0.003 |
| *Little¹* | 7.91 (6.36 - 8.73) |  |
| *Much²* | 7.55 (6.09 - 8.36) |  |

*¹nothing/very little/more or less. ²fairly/extremely. ³very poor/poor/neither bad nor good. ^4^good/very good.  ^5^nothing/very little/more or less. ^6^many times/always.*
