# Supplementary material for: An explorative analysis of the differences in levels of happiness between cancer patients, informal caregivers and the general population
Source: BMC Palliat Care. 2020 Jul 11;19:106. doi: 10.1186/s12904-020-00594-1 (PMC7354680; doi:10.1186/s12904-020-00594-1)
Supplement: Supplementary file 3 — Additional file 3: Supplementary Material 3. Univariate analysis for the evaluation of characteristics associated with satisfaction with life measured by the Satisfaction with Life Scale (SWLS) (n = 2580). Items used in the univariate analysis to assess the characteristics associated with satisfaction with life. [file 12904_2020_594_MOESM3_ESM.docx]

| **Supplementary Material 3 –** Univariate analysis for the evaluation of characteristics associated with satisfaction with life measured by Satisfaction with Life Scale (SWLS) (n=2580). | | |
| --- | --- | --- |
| **Variables** | **Median (P25 – P75)** | **p-Value** |
| Participants |  | <0.001 |
| *General population* | 26 (21-30) |  |
| *Caregivers of cancer patients* | 28 (24-31) |  |
| *Cancer patients* | 28 (23-32) |  |
| Age (years) |  | <0.001 |
| *18-29* | 26 (20-29) |  |
| *30-39* | 26 (21-30) |  |
| *40-49* | 27 (21-30) |  |
| *50-59* | 28 (23.5-31) |  |
| *60-69* | 28 (24-32) |  |
| *≥70* | 30 (27-34) |  |
| Marital status |  | <0.001 |
| *Married* | 28 (23-31) |  |
| *Windowed* | 26.5 (21.5-32) |  |
| *Separated or divorced* | 24 (17-29) |  |
| *Single* | 25 (20-29) |  |
| Educational level |  | <0.001 |
| *<8 years of education* | 29 (23-33) |  |
| *8 to 11 years of education* | 26 (19-30) |  |
| *>11 years of education* | 26 (21-30) |  |
| Family income* |  | <0.001 |
| *≤3.9 minimum wages* | 25 (18-30) |  |
| *≥4 minimum wages* | 27 (22-30) |  |
| Has current professional activity |  | <0.001 |
| *Yes* | 27 (21-30) |  |
| *No* | 27 (18-29) |  |
| Feeling of happiness with the professional activity |  | <0.001 |
| *Hasn’t professional activity* | 24 (17-29) |  |
| *Has professional activity* | 27 (22 – 30) |  |
| Place of residence (Brazilian region) |  | 0.046 |
| *Midwest* | 26 (20-30) |  |
| *Northeast* | 25 (19-30) |  |
| *North* | 26.5 (22-30) |  |
| *Southeast* | 27 (22-30) |  |
| *South* | 26 (21-30) |  |
| Location where live |  | 0.004 |
| *Urban Area* | 26 (21-30) |  |
| *Rural Area* | 28 (23-31) |  |
| Family Funding Program (“Bolsa Família”) |  | 0.007 |
| *No* | 27 (21-30) |  |
| *Yes* | 21 (16-28) |  |
| Religious beliefs |  | <0.001 |
| *Catholic* | 27 (22-30) |  |
| *Evangelic* | 26 (21-29.5) |  |
| *Spiritist* | 27 (22-30) |  |
| *Other* | 25.5 (19-31) |  |
| *Atheist / Agnostic / No formal religion* | 25 (19-29) |  |
| Voluntary activity |  | <0.001 |
| *No* | 26 (20-30) |  |
| *Yes* | 28 (23-31) |  |
| Voluntary financial donation |  | <0.001 |
| *No* | 26 (20-29) |  |
| *Yes* | 28 (23-31) |  |
| Cat as a pet |  | 0.003 |
| *No* | 27 (21-30) |  |
| *Yes* | 25 (20-29) |  |
| Self described as |  | <0.001 |
| *Pessimistic* | 19 (14-25) |  |
| *Neither optimistic nor pessimistic* | 23 (18-28) |  |
| *Optimistic* | 28 (24-31) |  |
| Current health problem |  | <0.001 |
| *Yes* | 26 (20-30) |  |
| *No* | 27 (22-30) |  |
| Diagnosis and treatment of current cancer |  | <0.001 |
| *No* | 26 (21-30) |  |
| *Yes* | 28 (23-32) |  |
| Diagnosis of depression |  | <0.001 |
| *No* | 27 (22-30) |  |
| *Yes* | 20 (14-25) |  |
| Diagnosis of anxiety |  | <0.001 |
| *No* | 27 (22-30) |  |
| *Yes* | 23 (16-28) |  |
| Diagnosis of panic disorder |  | <0.001 |
| *No* | 27 (22-30) |  |
| *Yes* | 19 (13-24) |  |
| Other psychological/psychiatric problem |  | <0.001 |
| *No* | 27 (21-30) |  |
| *Yes* | 22 (15.5-26) |  |
| Influence of religious or spiritual life on happiness |  | <0.001 |
| *Little¹* | 25 (18-29) |  |
| *Much²* | 28 (23-31) |  |
| Self-assessment of health |  | <0.001 |
| *Bad³* | 22 (16-28) |  |
| *Good* *^4^* | 27 (23-30) |  |
| Frequency of family gatherings |  | <0.001 |
| *Little ^5^* | 24 (19-28) |  |
| *Much^6^* | 28 (23-31) |  |
| Contact with nature |  | <0.001 |
| *Little ^5^* | 26 (20-30) |  |
| *Much^6^* | 29 (24-32) |  |
| Physical activity |  | <0.001 |
| *Don’t practice physical activity* | 26 (19-30) |  |
| *Once to twice per week* | 26 (21-30) |  |
| *3 or more times per week* | 28 (23-31) |  |
| Leisure time |  | <0.001 |
| *Little¹* | 25 (20-29) |  |
| *Much²* | 29 (25-31) |  |
| Feeling of happiness with the professional activity |  | <0.001 |
| *Little¹* | 23 (17-28) |  |
| *Much²* | 28 (24-31) |  |
| Satisfaction with financial issues |  | <0.001 |
| *Little¹* | 24 (18-28) |  |
| *Much²* | 29 (27-32) |  |
| Happiness affected by loved one's disease |  | <0.001 |
| *Little¹* | 27 (23-30) |  |
| *Much²* | 25.5 (20-30) |  |

*brazilian minimum wage.

*¹nothing/very little/more or less. ²fairly/extremely. ³very poor/poor/neither bad nor good. ^4^good/very good.  ^5^nothing/very little/more or less. ^6^many times/always.*
