# Supplementary material for: An explorative analysis of the differences in levels of happiness between cancer patients, informal caregivers and the general population
Source: BMC Palliat Care. 2020 Jul 11;19:106. doi: 10.1186/s12904-020-00594-1 (PMC7354680; doi:10.1186/s12904-020-00594-1)
Supplement: Supplementary file 4 — Additional file 4: Supplementary Material 4. Univariate analysis for the evaluation of characteristics associated with positive affect measured by Diener and Emmon’s Positive and Negative Experience Scale (PNES) (n = 2580). Items used in the univariate analysis to assess the characteristics associated with positive affect. [file 12904_2020_594_MOESM4_ESM.docx]

| **Supplementary Material 4 –** Univariate analysis for the evaluation of characteristics associated with positive affects measured by Diener and Emmon’s Positive and Negative Experience Scale (PNES) (n=2580). | | |
| --- | --- | --- |
| **Variables** | **Median (P25 – P75)** | **p-Value** |
| Participants |  | <0.001 |
| *General population* | 25 (20-28) |  |
| *Caregivers of cancer patients* | 22 (19-26) |  |
| *Cancer patients* | 21 (18-23) |  |
| Gender |  | <0.001 |
| *Male* | 25 (21-29) |  |
| *Female* | 24 (20-27) |  |
| Race |  | 0.049 |
| *White* | 24 (20-28) |  |
| *Black* | 23 (19-27) |  |
| *Latino* | 23 (19-27) |  |
| *Asian* | 25 (21-28) |  |
| Age (years) |  | <0.001 |
| *18-29* | 24 (20-28) |  |
| *30-39* | 24 (20-28) |  |
| *40-49* | 24 (20-28) |  |
| *50-59* | 22 (19-26) |  |
| *60-69* | 22 (20-25) |  |
| *≥70* | 23 (20-27) |  |
| Marital Status |  | <0.001 |
| *Married* | 24 (20-28) |  |
| *Windowed* | 21 (17-25) |  |
| *Separated or divorced* | 22 (18.5-26) |  |
| *Single* | 24 (20-28) |  |
| Educational Level |  | <0.001 |
| *<8 years of education* | 21 (18-24) |  |
| *8 to 11 years of education* | 23 (19-27) |  |
| *>11 years of education* | 24 (20-28) |  |
| Family income* |  | <0.001 |
| *≤3.9 minimum wages* | 22 (19-27) |  |
| *≥4 minimum wages* | 24 (20-28) |  |
| Has current professional activity |  | 0.032 |
| *Yes* | 24 (20-28) |  |
| *No* | 22 (19-26) |  |
| Feeling of happiness with the professional activity |  | 0.046 |
| *Hasn’t professional activity* | 23 (18-27) |  |
| *Has professional activity* | 24 (20-28) |  |
| Place of residence (Brazilian region) |  | 0.031 |
| *Midwest* | 23 (19-27) |  |
| *Northeast* | 25 (20-28) |  |
| *North* | 23 (20-27) |  |
| *Southeast* | 23 (20-28) |  |
| *South* | 25 (20-28) |  |
| Any Government Funding Program |  | <0.001 |
| *Yes* | 23 (19-26) |  |
| *No* | 24 (20-28) |  |
| Retirement due to disability |  | <0.001 |
| *No* | 24 (20-28) |  |
| *Yes* | 22 (17-24) |  |
| Sickness Funding Program (“Auxílio doença”) |  | <0.001 |
| *No* | 24 (20-28) |  |
| *Yes* | 21 (18-25) |  |
| Religious beliefs |  | 0.016 |
| *Catholic* | 24 (20-28) |  |
| *Evangelic* | 23 (19-27) |  |
| *Spiritist* | 24 (20-28) |  |
| *Other* | 24 (19.5-29) |  |
| *Atheist / Agnostic / No formal religion* | 23 (19-27) |  |
| Voluntary activity |  | <0.001 |
| *No* | 23 (20-27) |  |
| *Yes* | 25 (21-29) |  |
| Voluntary financial donation |  | <0.001 |
| *No* | 23 (19-27) |  |
| *Yes* | 25 (21-29) |  |
| Cat as a pet |  | 0.017 |
| *No* | 24 (20-28) |  |
| *Yes* | 23 (19-28) |  |
| Self described as |  | <0.001 |
| *Pessimistic* | 18 (15-21) |  |
| *Neither optimistic nor pessimistic* | 21 (18-25) |  |
| *Optimistic* | 25 (22-29) |  |
| Current health problem |  | <0.001 |
| *Yes* | 22 (19-26) |  |
| *No* | 25 (21-29) |  |
| Diagnosis and treatment of current cancer |  | <0.001 |
| *No* | 25 (20-28) |  |
| *Yes* | 21 (18-23) |  |
| Diagnosis of depression |  | <0.001 |
| *No* | 24 (20-28) |  |
| *Yes* | 19 (16-23) |  |
| Diagnosis of anxiety |  | <0.001 |
| *No* | 24 (20-28) |  |
| *Yes* | 22 (18-26) |  |
| Diagnosis of panic disorder |  | <0.001 |
| *No* | 24 (20-28) |  |
| *Yes* | 20 (16-25) |  |
| Other psychological/psychiatric problem |  | <0.001 |
| *No* | 24 (20-28) |  |
| *Yes* | 21 (16-23) |  |
| Influence of religious or spiritual life on happiness |  | <0.001 |
| *Little¹* | 23 (19-27) |  |
| *Much²* | 24 (20-28) |  |
| Self-assessment of health |  | <0.001 |
| *Bad³* | 20 (17-24) |  |
| *Good* *^4^* | 25 (21-28) |  |
| Frequency of family gatherings |  | <0.001 |
| *Little ^5^* | 23 (19-26) |  |
| *Much^6^* | 25 (21-29) |  |
| Contact with nature |  | <0.001 |
| *Little ^5^* | 24 (20-27) |  |
| *Much^6^* | 25 (21-29) |  |
| Physical activity |  | <0.001 |
| *Don’t practice physical activity* | 22 (19-26) |  |
| *Once to twice per week* | 24 (20-28) |  |
| *3 or more times per week* | 25 (21-29) |  |
| Leisure time |  | <0.001 |
| *Little¹* | 22 (19-26) |  |
| *Much²* | 27 (23-30) |  |
| Feeling of happiness with the professional activity |  | <0.001 |
| *Little¹* | 21 (18-25) |  |
| *Much²* | 25 (21-29) |  |
| Satisfaction with financial issues |  | <0.001 |
| *Little¹* | 22 (19-26) |  |
| *Much²* | 26 (23-29) |  |
| Happiness affected by loved one's disease |  | <0.001 |
| *Little¹* | 25 (21-29) |  |
| *Much²* | 23 (20-27) |  |

***brazilian minimum wage.

*¹nothing/very little/more or less. ²fairly/extremely. ³very poor/poor/neither bad nor good. ^4^good/very good.  ^5^nothing/very little/more or less. ^6^many times/always.*
