# Supplementary material for: An explorative analysis of the differences in levels of happiness between cancer patients, informal caregivers and the general population
Source: BMC Palliat Care. 2020 Jul 11;19:106. doi: 10.1186/s12904-020-00594-1 (PMC7354680; doi:10.1186/s12904-020-00594-1)
Supplement: Supplementary file 5 — Additional file 5: Supplementary Material 5. Univariate analysis for the evaluation of characteristics associated with negative affect measured by Diener and Emmon’s Positive and Negative Experience Scale (PNES) (n = 2580). Items used in the univariate analysis to assess the characteristics associated with negative affect. [file 12904_2020_594_MOESM5_ESM.docx]

| **Supplementary Material 5 –** Univariate analysis for the evaluation of characteristics associated to negative affects measured by Diener and Emmon’s Positive and Negative Experience Scale (PNES) (n=2580). | | |
| --- | --- | --- |
| **Variables** | **Median (P25 – P75)** | **p-Value** |
| Participants |  | <0.001 |
| *General population* | 12 (09-16) |  |
| *Caregivers of cancer patients* | 16 (14-19) |  |
| *Cancer patients* | 15 (13-17) |  |
| Age (years) |  | 0.012 |
| *18-29* | 13 (10-16) |  |
| *30-39* | 13 (10-16) |  |
| *40-49* | 13 (10-16) |  |
| *50-59* | 13 (10-16) |  |
| *60-69* | 14 (12-17) |  |
| *≥70* | 14 (12-17) |  |
| Marital Status |  | 0.005 |
| *Married* | 13 (10-16) |  |
| *Windowed* | 14 (12-18) |  |
| *Separated or divorced* | 14 (11-16) |  |
| *Single* | 13 (10-17) |  |
| Educational Level |  | <0.001 |
| *<8 years of education* | 16 (13-18) |  |
| *8 to 11 years of education* | 14 (11-17) |  |
| *>11 years of education* | 12 (10-16) |  |
| Family income* |  | <0.001 |
| *≤3.9 minimum wages* | 15 (11-18) |  |
| *≥4 minimum wages* | 12 (10-16) |  |
| Has current professional activity |  | 0.004 |
| *Yes* | 13 (10-16) |  |
| *No* | 15 (11-17) |  |
| Place of residence (Brazilian region) |  | <0.001 |
| *Midwest* | 14 (11-17) |  |
| *Northeast* | 13 (09-16) |  |
| *North* | 14 (11-16) |  |
| *Southeast* | 13 (10-16) |  |
| *South* | 12 (09-16) |  |
| Government aid |  | <0.001 |
| *Yes* | 14 (11-17) |  |
| *No* | 13 (10-16) |  |
| Retirement due to disability |  | <0.001 |
| *No* | 13 (10-16) |  |
| *Yes* | 15 (12-17) |  |
| Sickness Funding Program (“Auxílio doença”) |  | <0.001 |
| *No* | 13 (10-16) |  |
| *Yes* | 15 (12-18) |  |
| Family Funding Program (“Bolsa-família”) |  | 0.001 |
| *No* | 13 (10-16) |  |
| *Yes* | 15 (13-19) |  |
| Self described as |  | <0.001 |
| *Pessimistic* | 17 (14-20) |  |
| *Neither optimistic nor pessimistic* | 15 (12-17) |  |
| *Optimistic* | 12 (09-15) |  |
| Current health problem |  | <0.001 |
| *Yes* | 14 (11-17) |  |
| *No* | 12 (09-15) |  |
| Diagnosis and treatment of current cancer |  | <0.001 |
| *No* | 13 (10-16) |  |
| *Yes* | 15 (13-17) |  |
| Diagnosis of depression |  | <0.001 |
| *No* | 13 (10-16) |  |
| *Yes* | 17 (14-20) |  |
| Diagnosis of anxiety |  | <0.001 |
| *No* | 13 (10-16) |  |
| *Yes* | 15 (12-18) |  |
| Diagnosis of panic disorder |  | <0.001 |
| *No* | 13 (10-16) |  |
| *Yes* | 17 (12-20) |  |
| Other psychological/psychiatric problem |  | <0.001 |
| *No* | 13 (10-16) |  |
| *Yes* | 16 (12-19) |  |
| Leisure time |  | <0.001 |
| *Little¹* | 14 (11-17) |  |
| *Much²* | 11 (08-15) |  |
| Self-assessment of health |  | <0.001 |
| *Bad³* | 15 (13-19) |  |
| *Good* *^4^* | 12 (10-16) |  |
| Frequency of family gatherings |  | <0.001 |
| *Little ^5^* | 14 (11-17) |  |
| *Much^6^* | 13 (10-16) |  |
| Contact with nature |  | 0.005 |
| *Little ^5^* | 13 (10-17) |  |
| *Much^6^* | 13 (10-16) |  |
| Physical activity |  | <0.001 |
| *Don’t practice physical activity* | 14 (11-17) |  |
| *Once to twice per week* | 13 (10-16) |  |
| *3 or more times per week* | 12 (09-15) |  |
| Feeling of happiness with the professional activity |  | <0.001 |
| *Little¹* | 15 (11-18) |  |
| *Much²* | 12 (09-15) |  |
| Satisfaction with financial issues |  | <0.001 |
| *Little¹* | 14 (11-17) |  |
| *Much²* | 12 (09-15) |  |
| Sickness in a close person (a loved one) |  | <0.001 |
| *Yes* | 14 (11-17) |  |
| *No* | 13 (10-16) |  |
| Happiness affected by loved one's disease |  | <0.001 |
| *Little¹* | 12 (09-16) |  |
| *Much²* | 14 (12-17) |  |

*brazilian minimum wage.

*¹nothing/very little/more or less. ²fairly/extremely. ³very poor/poor/neither bad nor good. ^4^good/very good.  ^5^nothing/very little/more or less. ^6^many times/always.*
